# Outcomes in extremely low‐birthweight infants: What can we learn by comparing epidemiological studies over time?

**DOI:** 10.1111/ppe.12922

**Published:** 2022-08-23

**Authors:** Véronique Pierrat, Pierre‐Yves Ancel

**Affiliations:** ^1^ CRESS, Obstetrical Perinatal and Pediatric Epidemiology Research Team, EPOPé, INSERM, INRAE Université Paris Cité Paris France; ^2^ CHU Lille, Department of Neonatal Medicine Jeanne de Flandre Hospital Lille France; ^3^ Center for Clinical Investigation P1419, APHP APHP Centre‐ Université Paris Cité Paris France

Since the introduction of care to neonates born extremely preterm (EP) or with extremely low birthweight (ELBW), survival and survival without neurodevelopmental disabilities in this high‐risk population have dramatically improved in high‐income countries.^1^ Trends in care practices, morbidity, and mortality for these EP infants have been reported over the past three decades, after the wide implementation of antenatal steroids and surfactant.^2^ Changes have been observed in maternal and infant care practices, and these have been associated with a decrease in mortality and in several neonatal morbidities.^1^, ^2^

In this issue of *Paediatric and Perinatal Epidemiology*, Zayegh and colleagues^3^ reported on trends in survival, perinatal morbidity, and 2‐year neurodevelopmental outcomes in ELBW infants over four decades. They used data from six geographic cohorts collected through the Victorian Infant Collaborative Study (VICS) research program in Australia with children born between 1979 and 2017 and with a birthweight of 500–999 g. The VICS project is unique in the field of neonatology. Strong design features, including the selection of complete geographic cohorts, reference cohorts of normal birthweight, and term‐born live births to provide contemporary outcomes in non‐EP birth/non‐ELBW children, and face‐to‐face long‐term assessment by blinded assessors, were used from the onset and in successive cohorts allowing longitudinal analyses across different eras. As already described,^1^, ^2^ Zayegh et al^3^ reported that active care and survival rates of ELBW have increased dramatically, but with modest reductions in neonatal morbidity and even an increase in bronchopulmonary dysplasia. At the same time, the absolute numbers of survivors with major disability per year rose from 12.5 in 1979–1980 to 30 in 2016–2017 but the absolute numbers of survivors free of major disability rose much more, from 31 per year in 1979–1980 to 147 in 2016–2017. Overall, results suggest that the increase in survival observed between the six periods was associated with relative stability of major disability among survivors.

Although in agreement with findings comparing cohorts over time in other high‐income countries,^1^, ^2^ a unique contribution of this study is that it covers a much larger time span, with the first two cohorts established before the use of exogenous surfactant and the extensive adoption of antenatal steroids and the next four covering changes in care practices such as the use of surfactant, ethical attitudes towards care for ELBW infants, the physical environment of the NICUs, staffing, new modes of oxygenation, extensive use of non‐invasive ventilation, and better implementation of evidence‐based care.^4^ This temporality makes it possible to better appreciate the evolutions and fluctuations over time and to analyse not only improvements in health but also outcomes that are stable or evolve with non‐linear trends. Finally, putting together data collected over such an extended time period may identify individual characteristics associated with outcomes that are not related to care practices and in situations that are too rare to be analysed using shorter time windows.

The inclusion of cohorts dating back to the late 1970s necessitated the selection of infants on the basis of birthweight rather than gestational age since gestational was less reliable and not used for the cohorts established in the 1970/1980s. This could be seen as a limitation of the study, however, analysis in more detail of birthweight *z*‐scores suggests that fetal growth restriction was unlikely to have affected the results. In addition, a high overlap between gestational age and birthweight has been observed, especially in EP or ELBW populations, and yields similar results when considering long‐term outcomes.^5^ Reporting neurodevelopmental outcomes before 3 years of age is another limitation. Results from the EPICure study^6^ (births at 22–25 weeks' GA in the United Kingdom and Ireland in 1995) and from the EXPRESS study^7^ (births at 22–26 weeks' GA in Sweden in 2004–2007) have shown that severe neurodevelopmental impairment at 2.5 years has good specificity but low sensitivity for diagnosing moderate or severe disability at 6–6.5 years. Among children assessed at both ages and classified as having a severe neurodevelopmental impairment at 2.5 years, 86% (54/63) in the EPICure study and 88% (40/45) in the EXPRESS study had moderate to severe neurodevelopmental impairment at 6–6.5 years. Conversely, 24% of infants classified as being free of neurodevelopmental impairment in the EPICure study and 23% of those with no or mild neurodevelopmental impairment at 2.5 years in the EXPRESS study had moderate to severe neurodevelopmental impairment at 6–6.5 years.

Nevertheless, what lessons does this study provide regarding the factors that contribute to or hinder change, or about the focus for practice change or research? Among organisational factors, the stability of inborn patients since 1991, apart from a substantial decrease in 1997 that was not sustained in 2005 and 2016–2017, is worth considering. In another study, the VICS group was able to show that maternal characteristics such as being a teen mother, a multigravida, or having a previous history of preterm birth were associated with a higher risk of delivery outside a perinatal center.^8^ This suggests that alongside the organisation of neonatal care taking into account regionalisation and medicalised neonatal transport, focusing prevention on the needs of these specific populations should be considered to decrease the number of outborn ELBW neonates. Apart from organisational factors, the evolution of neonatal morbidities is interesting to consider. Necrotising enterocolitis (NEC) and grade 3 or 4 intraventricular haemorrhage are reported to be “relatively stable.” Yet, there were marked fluctuations in the rates of NEC observed during the 40 years period suggesting potential non‐linear trends. The complications of NEC can have devastating health consequences and a better understanding of effective preventive strategies is urgently needed. However, due to small numbers, investigating risk factors for NEC will only be possible with larger cohorts or with international comparisons. The International Network for Evaluation of Outcomes of Neonates has shown that variability in surgical NEC was not associated with the implementation of prevention practices such as probiotics, feeding, or donor milk.^9^ The time required to implement better practices and the maintenance of these practices over time are poorly explored in the literature and could lead to non‐linear trends. The Zayegh et al^3^ report invites us to explore in more depth these different aspects of care.

Finally, the longitudinal approach over such a long period of time also makes it possible to analyse the evolution of variables that have an impact on family's life and health resources, such as the number of days before discharge. During the four decades period, the number of days before the discharge of these ELBW infants was relatively stable, from 97 days in 1978–1980 to 103 in 2016–2017, with an increase of 1 week that can be due to the decrease in mean gestational age from 27.1 to 26.4 weeks. For families, the burden of prolonged hospitalisation is high. Sustaining family‐centred care together with developmental care with the aim to empower parents as partners in care for their prematurely born baby is increasingly acknowledged by parental groups of preterm‐born neonates to enhance future outcomes for children and families.^10^ Family‐centered care has been shown to have an impact on the length of stay^10^ and the results reported by Zayegh et al^3^ illustrate the need for efforts to implement these strategies.

Although there have been extensive publications from the VICS group, this descriptive and longitudinal approach, including cohorts born before the 1990s, adds new insights into the improvement of care for infants born EP or with ELBW. Neonatal morbidities that were the most interesting to consider for further improvement were those that were described as “relatively stable” over time, or with fluctuations that could be worth better understanding. They could help to draw new hypotheses and new analysis strategies to improve the care of EP and ELBW neonates.

## AUTHOR CONTRIBUTIONS

Dr Pierrat and Pr Ancel both contributed to the analysis of the Zayegh paper. Dr Pierrat wrote the commentary and Pr Ancel approved the final version of the manuscript.

## About the authors

**Véronique Pierrat** is a neonatologist in Lille (France) and researcher in the Obstetrical, Perinatal and Paediatric Epidemiology Research Team (EPOPé), Paris (France). Dr Pierrat completed his doctoral training at the University of Utrecht (The Netherlands) under the supervision of Pr Linda Simona de Vries.

**Pierre‐Yves Ancel** is a professor of epidemiology and public health. He is a team leader of the “Obstetrical, Perinatal and Pediatric Epidemiology Team (EPOPé), Inserm 1153, Paris (France)” and coordinator of the “Center for Clinical Investigation P1419, Cochin – Port‐Royal Hospital, Paris (France).” Both have research focusing on assessing the long‐term consequences of preterm births and their perinatal determinants mainly based on the French Epipage cohorts. They are also involved in the EPICE/SHIPS project coordinated by Pr Jennifer Zeitlin on the effects of differences in attitudes of care on survival, morbidity, and development of children born at extremely low gestational ages in France and other European countries.

## CONFLICTS OF INTEREST

Dr Pierrat and Pr Ancel have no disclosures or conflicts of interest to declare.

## Data Availability

Not applicable for this commentary.
